# An Eye in the Replication Stress Response: Lessons From Tissue-Specific Studies *in vivo*

**DOI:** 10.3389/fcell.2021.731308

**Published:** 2021-11-04

**Authors:** Gabriel E. Matos-Rodrigues, Rodrigo A. P. Martins

**Affiliations:** Programa de Biologia Celular e do Desenvolvimento, Instituto de Ciências Biomédicas, Universidade Federal do Rio de Janeiro, Rio de Janeiro, Brazil

**Keywords:** genome stability, cell cycle, DNA damage, chekcpoint, ATR, organogenesis, retina, lens

## Abstract

Several inherited human syndromes that severely affect organogenesis and other developmental processes are caused by mutations in replication stress response (RSR) genes. Although the molecular machinery of RSR is conserved, disease-causing mutations in RSR-genes may have distinct tissue-specific outcomes, indicating that progenitor cells may differ in their responses to RSR inactivation. Therefore, understanding how different cell types respond to replication stress is crucial to uncover the mechanisms of RSR-related human syndromes. Here, we review the ocular manifestations in RSR-related human syndromes and summarize recent findings investigating the mechanisms of RSR during eye development *in vivo*. We highlight a remarkable heterogeneity of progenitor cells responses to RSR inactivation and discuss its implications for RSR-related human syndromes.

## Introduction

Maintenance of genome stability is essential for development and homeostasis, and failures in processes required for genomic stability are associated with various human syndromes (Ciccia and Elledge, 2010; Negrini et al., 2010; O’Driscoll, 2012). DNA replication, transcriptional regulation and chromatin modifications must be precisely coordinated to ensure faithful transmission of genetic information to stem/progenitor cell pools that expand during development (Prioleau and MacAlpine, 2016). During DNA synthesis, many sources of genotoxic stress may slow or stall the progression of replication forks, a condition defined as replication stress. As a consequence, cells trigger the replication stress response (RSR). Activation of the RSR signaling pathways may slow DNA replication and allow extra time for DNA repair, preventing DNA mutations, chromosomal rearrangements and, therefore, genomic instability (Zeman and Cimprich, 2014; Techer et al., 2017; Tubbs and Nussenzweig, 2017). Due to its essential role during replication and development, mutations in genes that code proteins required for RSR are associated with several developmental syndromes (Zeman and Cimprich, 2014; Munoz and Mendez, 2017). Here, we review the ocular manifestations in RSR-related human syndromes and discuss recent findings investigating tissue-specific RSR in the developing eye that may contribute to understanding how defective-RSR drives developmental malformations.

## Replication Stress Response

**FIGURE 1:**
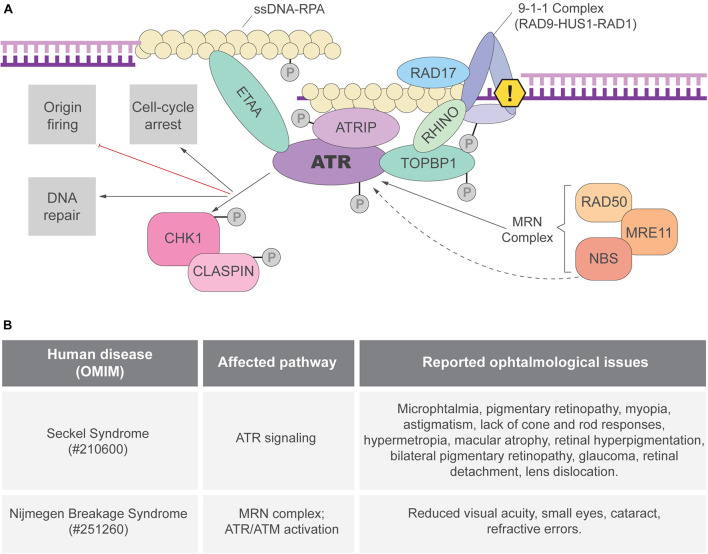
ATR activation and the replication stress response (RSR). **(A)** The ATRIP-ATR complex is recruited to RPA coated ssDNA. ATR can be directly activated by TOPBP1 or ETAA1 *via* their AAD domain. In regions of dsDNA-ssDNA junctions, the 9-1-1 complex is responsible for TOPBP1 recruitment and ATR activation. ETAA1-mediated ATR activation is not dependent on ssDNA-dsDNA junctions as ETAA1 directly binds to RPA-coated ssDNA. RHINO and the MRN complex are also important for ATR activation that phosphorylates different targets, including the CHK1 kinase. Once the RSR is activated, ATR and its downstream targets can modify different aspects of cell metabolism to prevent genome instability. **(B)** Ocular manifestations reported in patients of the RSR-related syndromes: Seckel (Lim and Wong, 1973; Guirgis et al., 2001; Reddy and Starr, 2007; Aktas et al., 2013; Krzyzanowska-Berkowska et al., 2014) and Nijmegen breakage syndromes (Varon et al., 1998; Gralek et al., 2011).

Single-stranded DNA (ssDNA) breaks are proposed to be the most frequent DNA lesion (∼75%) and those are normally generated during DNA replication (Lindahl and Barnes, 2000; Tubbs and Nussenzweig, 2017). The formation ssDNA stretches and aberrant replication fork structures lead to the activation of the ATR kinase, the master regulator of the RSR (Figure 1A). When exposed, long ssDNA stretches are coated by the replication protein A (RPA) complex. ATR-interacting protein (ATRIP), a mutually dependent partner of ATR, directly binds to RPA and recruits ATR to the RPA-ssDNA sites (Hekmat-Nejad et al., 2000; Cortez et al., 2001; Zou and Elledge, 2003; Dart et al., 2004; Ball et al., 2005) (Figure 1A). ATR recruitment is not sufficient for its full activation and many regulatory partners are necessary (Saldivar et al., 2017). In double-stranded DNA-ssDNA (dsDNA-ssDNA) junctions, such as the ones found in stalled replication forks, ATR activation requires DNA topoisomerase II-binding protein 1 (TOPBP1) (Kumagai et al., 2006). TOPBP1 recruitment to dsDNA-ssDNA junctions depends on its interaction with RAD9, member of the 9-1-1 clamp complex (RAD9-RAD1-HUS1) that is recruited by the clamp load factor RAD17 (Bermudez et al., 2003; Kumagai et al., 2006; Delacroix et al., 2007) (Figure 1A). TOPBP1 recruitment depends on other proteins, including the MRE11-RAD50-NBS1 (MRN) complex and RHINO (Cotta-Ramusino et al., 2011; Duursma et al., 2013). Importantly, NBS1 and the MRN complex are directly involved in ATR activation and cells from patients with inactivating mutations in *NBS1* exhibit defective RSR (Stiff et al., 2005; Duursma et al., 2013; Shiotani et al., 2013). In ssDNA regions without ssDNA-dsDNA junctions, RSR activation can be mediated by ETAA1, that directly interacts with RPA and activates ATR through its ATR-activating domain (AAD) domain (Figure 1A; Bass et al., 2016; Haahr et al., 2016; Lee et al., 2016; Thada and Cortez, 2019). Studies in human cell lines suggested that ATR activation by TOPBP1 and ETAA1 may occur in different contexts. TOPBP1 would activate ATR upon induced replication stress and ETAA1 would trigger ATR activation in unchallenged replication to avoid under-replicated DNA during the S-M transition (Saldivar et al., 2018). In addition, ATR can be directly activated by NBS1, although the mechanisms are not clear since NBS1 does not have an AAD domain (Kobayashi et al., 2013).

RSR depends not only on ATR-mediated signal transduction but also on its downstream effectors, specially the checkpoint protein 1 (CHK1) (Saldivar et al., 2017). ATR phosphorylates CHK1 in multiple sites and CHK1 activation depends on its partner CLASPIN (Kumagai and Dunphy, 2000; Liu et al., 2000, 2006; Zhao and Piwnica-Worms, 2001; Figure 1A). Once activated, the ATR-CHK1 signaling triggers local (e.g., dormant replication fork firing) and global (e.g., cell cycle arrest) responses to ensure the faithful duplication of the genome (Saldivar et al., 2017).

## Inactivation of the Replication Stress Response *in vivo*

Highlighting the importance of ATR activation for unchallenged cell proliferation during development *in vivo*, inactivation of various “RSR genes” (here defined as genes necessary for full activation of ATR-CHK1 signaling following replication stress) is embryonic lethal in mice (Luo et al., 1999; Brown and Baltimore, 2000; de Klein et al., 2000; Liu et al., 2000; Weiss et al., 2000; Zhu et al., 2001; Dumon-Jones et al., 2003; Budzowska et al., 2004; Hopkins et al., 2004; Wang et al., 2005; Han et al., 2010; Jeon et al., 2011; Yang et al., 2016; Miosge et al., 2017). Although RSR has been extensively studied in various models, the mechanisms of the ATR activation and, therefore, the exact roles of ATR regulators in unchallenged replication *in vivo* are still not completely understood. For example, it was clear that ATR protein stability and function depend on its interaction with ATRIP in human cells (Cortez et al., 2001), however, prior to our recent work (Matos-Rodrigues et al., 2020a,b) ATRIP function had not been investigated *in vivo*. Moreover, while ETAA1 plays an essential role in an ATR-regulated S-G2 checkpoint in immortalized cells (Saldivar et al., 2018), ETAA1 null mice show a mild phenotype of partial embryonic lethality (Miosge et al., 2017). In contrast, ATR activation by TOPBP1 has an essential role in unchallenged replication *in vivo*, since disruption of ATR activation by TOPBP1 leads to embryonic lethality in mice (Zhou et al., 2013). These data indicate that ATR activation by TOPBP1, but not ETAA1, is essential for unchallenged replication in mice. The reason behind these distinct requirements in cultured human cells and in mouse development remains unclear.

## Replication Stress Response *in vivo*: Focus on the Eye

**FIGURE 2:**
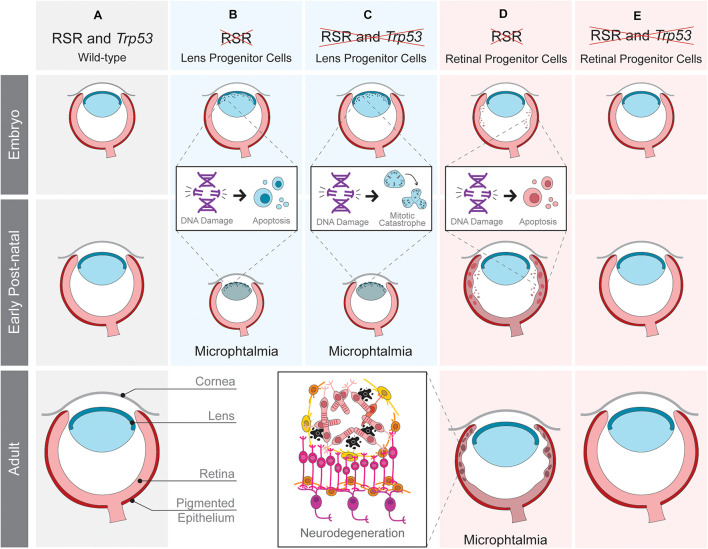
Tissue-specific effects of replication stress response (RSR) inactivation in developing mouse eye. Schematic representation of ocular development in wild-type mice **(A)** and the consequences of RSR inactivation in lens **(B,C)** or retinal progenitor cells **(D,E)** in *Trp53-*proficient **(B,D)** and *Trp53-*deficient scenarios **(C,E)**. RSR inactivation by the loss of ATRIP leads to progenitor cell apoptosis in both the lens and retina. Only in the retina, *Trp53*-deficiency rescued embryonic apoptosis and the consequent secondary phenotypes.

The eye is the sensory organ responsible for vision and is composed of three main tissues: cornea, lens and retina (Figure 2A). The anterior segment of the eye comprises the cornea, the iris and the lens, a transparent structure that focus the light to the back of the eye. The main tissue of its posterior segment is the retina, the neural part of the eye responsible for detection and preprocessing of the visual stimuli before transmission to the visual centers of the brain through the optic nerve (Dowling, 1987). The development of these ocular tissues is extremely interdependent. In mice, on the ninth day of embryonic development (E9), a projection of the diencephalon, the optic vesicle, encounters the surface ectoderm of the head and starts eye organogenesis by triggering the invagination of both structures. While the invagination of the surface ectoderm gives rise to the lens, the retina originates from the invaginating optic vesicle (Miesfeld and Brown, 2019).

Importantly, the eye represents a unique model to study the impact of defective RSR to organogenesis because: (1) of the vast knowledge about its development in mammals; (2) it is a non-essential organ, therefore a powerful model to analyze genetic interactions, and evaluate the long term consequences of essential genes inactivation; (3) there is a substantial amount of genetic tools available; (4) it is composed of tissues derived from distinct developmental lineages, making it ideal to study progenitor cells of different lineages. In addition, although clinical studies have shown ophthalmological manifestations in RSR-related syndromes (Figure 1B), the origins of these manifestations in these syndromes have been underexplored and raising awareness to this topic may bring important contributions to patients.

Loss-of-function mutations in ATR/ATRIP or in NBS1 are among the known causes of Seckel or Nijmegen breakage syndrome, respectively. These syndromes are characterized by moderate to severe tissue-growth impairments, neurodevelopmental defects and a series of ocular manifestations that have been reported in patients (Lim and Wong, 1973; Varon et al., 1998; Guirgis et al., 2001; Reddy and Starr, 2007; Gralek et al., 2011; Aktas et al., 2013; Krzyzanowska-Berkowska et al., 2014). Due to the recent advances on the understanding of these genes in eye development, we focus on their functions and its related human syndromes.

## Ocular Manifestations in Replication Stress-Related Human Syndromes

### Microphthalmia

Microphthalmia is a disorder characterized by abnormally small eyes that display high genetic heterogeneity and may occur as part of a syndrome. Disproportional ocular growth may contribute to microphthalmia, since microphthalmic eyes are more affected in the posterior segment than the anterior (Verma and Fitzpatrick, 2007). Microphthalmia has been reported in both Seckel and Nijmegen breakage syndromes (Figure 1B). Studies in animal models (discussed in the next sections) suggested that defective cell proliferation and increased cell death may be the cause of microphthalmia following the inactivation of RSR genes (Yang et al., 2006; Rodrigues et al., 2013; Matos-Rodrigues et al., 2020a,b). However, the mechanisms driving eye growth defects in syndromes caused by mutations in RSR-genes are far from being completely understood.

### Cataract

Although treatable, cataracts are the most common cause of blindness. Congenital cataracts, the ones in which the opacification of the lens is detected at birth, are a clinical feature of almost 200 syndromic genetic diseases (Liu et al., 2017; Berry et al., 2020). Many evidences directly associates cataractogenesis and DNA damage. Increased DNA oxidation has been found in cataract patients and is thought to trigger cataractogenesis (Osnes-Ringen et al., 2013; Zhang et al., 2014; Erol Tinaztepe et al., 2017; Uwineza et al., 2019). DNA repair genes are known risk factors for cataract (Su et al., 2013; Cui et al., 2017; Yang et al., 2018) and cataracts have been reported in Seckel syndrome patients (Rao et al., 2011) (Figure 1B). Human lens progenitor cells from cataract patients display increased levels of DNA single strand breaks, a hallmark of replication stress (Kleiman and Spector, 1993). Finally, sources of replication stress, such as oxidative damage, UV-light and ionizing radiation cause cataract (Liu et al., 2017). As expected, induced DNA damage disturbs the proliferation and differentiation of lens progenitor cells, which is proposed to be an underlying cause of ionizing radiation induced cataract (Uwineza et al., 2019). The molecular mechanisms driving these processes are still to be determined.

### Retinal Neurodegeneration

Glaucoma is characterized by structural damage to the optic nerve and retinal ganglion cell degeneration, leading to loss of vision due to the interruption of the transmission of information from the eye to the brain (Quigley, 2011; Calkins, 2012; Gemenetzi et al., 2012). Other retinopathies leading to neurodegeneration and vision loss include macular degeneration, retinopathy diabetic and retinitis pigmentosa (Massengill et al., 2018). Glaucoma, photoreceptors degeneration and lack of photoreceptor electrical responses were reported in patients with Seckel syndrome (Figure 1B). Importantly, replication stress has also been associated with the activation of pro-inflammatory pathways, which might fuel retinal neurodegeneration (Charlier and Martins, 2020; Ragu et al., 2020).

## Lessons From Mouse Models

Genetic inactivation of NBS1 in mice was key to understanding the etiology of Nijmegen breakage syndrome (Frappart and McKinnon, 2008). While NBS1 knockout in mice led to early embryonic lethality (Zhu et al., 2001), neural tissue-specific inactivation of NBS1 resulted in abnormalities similar to patients including microcephaly, growth retardation, cerebellar defects and ataxia (Frappart et al., 2005). Importantly, NBS1 loss in the developing brain led to distinct outcomes depending on the progenitor cell affected. For example, NBS1 deficiency in progenitor cells of the neocortex induced cell cycle arrest. In the cerebellum, growth defects are driven by progenitor cell death (Frappart et al., 2005; Li et al., 2012; Rodrigues et al., 2013).

In the developing eye, NBS1-deficiency in the lens leads to cell death, proliferation defects and microphthalmia (Yang et al., 2006; Baranes et al., 2009; Rodrigues et al., 2013). During retinogenesis, NBS1 is also required for retinal progenitor cell survival, but its inactivation does not affect eye growth (Rodrigues et al., 2013), most likely due to a minor contribution of retinal growth to eye size. Finding that NBS1 loss led to microphthalmia only when inactivated in lens progenitor cells provided a first hint of how RSR inactivation could affect eye development in a tissue-specific manner (Yang et al., 2006; Rodrigues et al., 2013). Interestingly, NBS1-deficient mature retinas undergo degeneration of the optic nerve and loss of retinal function (Baranes et al., 2009), but the molecular and cellular mechanisms underlying this neurodegeneration remain unclear.

Interestingly, a specific synergy between NBS1 loss and TRP53 was also revealed in lens progenitor cells. In the developing brain, TRP53 inactivation rescues cell death and proliferation defects and brain growth defects caused by NBS1 loss (Frappart et al., 2005; Li et al., 2012). In the lens, however, *Trp53* inactivation rescued progenitor cell death caused by NBS1 loss, but it did not rescue the defects in eye growth or cataract (Yang et al., 2006). Therefore, in NBS1*-*deficient lens progenitors, cell proliferation is blocked even when TRP53 is not functional, but the underlying mechanisms are still unknown. Importantly, in addition to its roles in RSR, NBS1 also participates in double-strand break signaling (Lee and Paull, 2005; Syed and Tainer, 2018), which could also factor in the diversity of outcomes observed.

Recently, we explored the function of another RSR gene by analyzing the function of ATRIP following tissue-specific inactivation in mice (Figure 2). As shown in transformed human cells (Cortez et al., 2001), ATR protein stability also depends on ATRIP in embryonic neural progenitor cells (Matos-Rodrigues et al., 2020a). Nestin-Cre-mediated inactivation of ATRIP in the developing central nervous system and in the eye leads to tissue growth defects (microphthalmia and microcephaly) that mirror the ones observed upon *Atr* inactivation (Lee et al., 2012). To understand the mechanisms underlying microphthalmia caused by ATRIP loss, we evaluated its contribution to cell cycle progression in *Trp53*-proficient and *Trp53*-deficient lens progenitor cells. In the presence of *Trp53*, ATRIP loss increases DNA damage and cell death, while in *Trp53*-deficient progenitors, ATRIP loss does not increase cell death, but leads to mitotic DNA damage and mitotic defects (Matos-Rodrigues et al., 2020a). These data suggest that inactivation of both genes might confer the ability to bypass the TRP53-mediated checkpoint and avoid cell death in S-phase, but ultimately culminating in mitotic catastrophe. Finally, as observed for NBS1, TRP53 deficiency does not rescue the microphthalmia caused by *Atrip* inactivation in lens progenitor cells.

We have also evaluated the effects of RSR inactivation in the mouse retina. ATRIP loss in embryonic retinal progenitor cells induces DNA damage accumulation and cell death, leading to lamination defects, photoreceptor degeneration and loss of vision (Matos-Rodrigues et al., 2020b). A previous study revealed photoreceptor degeneration in mice carrying an *Atr* hypomorphic mutation (Valdes-Sanchez et al., 2013). A role of ATR in the photoreceptor cilia was suggested to explain the observed neurodegeneration. Importantly, we found no evidence for a role of ATRIP in photoreceptors, since inactivation of *Atrip* specifically in these post-mitotic neurons did not affect retinal morphology or function. Because ATRIP is essential for ATR stability and all of its known functions are interdependent, further research is required to define the possible roles of the ATR-ATRIP complex in post-mitotic photoreceptor neurons.

In contrast to the lens, inactivation of *Trp53* rescues the cell death of retinal progenitor cells, neurodegeneration and visual impairment caused by ATRIP loss, indicating that TRP53-dependent apoptosis is the driver of retinal malformations caused by *Atrip* inactivation (Matos-Rodrigues et al., 2020b). These findings reinforced the existence of tissue-specific effects of RSR inactivation in the developing eye. An intact RSR is essential for lens progenitor cell proliferation since *Atrip* inactivation in the lens either abolishes lens formation (aphakia) or causes microphthalmia (Matos-Rodrigues et al., 2020a). In retinal progenitor cells, *Atrip* inactivation also leads to DNA damage accumulation and cell death. However, retinal development is not completely impaired by the slight modifications in proliferation and differentiation caused by defective RSR (Matos-Rodrigues et al., 2020b). These results suggest that lens progenitor cells are more sensitive to RSR inactivation than retinal ones and point to a different synergy between *Atrip* and *Trp53* when comparing retinal and lens progenitors. *Trp53* inactivation rescues lens progenitor cells apoptosis, but does not rescue eye growth defects, which were likely caused by enhanced mitotic DNA damage and mitotic defects (Matos-Rodrigues et al., 2020a). In opposition, *Trp53* inactivation completely rescues the developmental defects and the consequent neurodegeneration of the *Atrip*-deficient retinas (Figure 2). These observations are in agreement with previous data on the effects of NBS1 inactivation during mouse eye development.

## Discussion

Based on the above-described studies we propose that the eye growth defects observed in replication-stress related syndrome patients are caused by the essential function of the affected genes in RSR in progenitor cells during embryogenesis. For example, tissue dysplasia and photoreceptor degeneration observed in *Atrip*-deficient retinas are a secondary consequence of progenitor apoptosis caused by the defective RSR in progenitor cells during embryonic development (Matos-Rodrigues et al., 2020b). Reports of retinal malformations and degeneration have been found in Seckel and Nijmegen breakage syndrome (Figure 1B). However, possible non-canonical functions of RSR genes in post-mitotic cells should not be overlooked, as it has been recently shown that ATR-CHK1 pathway can have a direct function on post-mitotic neurons activity and regeneration in model organisms (Kirtay et al., 2021; Li et al., 2021). Clinical investigations performing follow up in RSR-related syndromes patients associated with molecular diagnosis can bring important insights on the eye manifestations of these disorders.

The DDR is an evolutionarily conserved process that is often believed to operate by universal uniform principles. However, given that different progenitor cells have distinct transcriptional programs, metabolism, microenvironment and face different DNA-damaging insults, the DDR presents cell type- and developmental stage-specific adaptations (Blanpain et al., 2011; Rodrigues et al., 2013; Kafer and Cesare, 2020). The heterogeneous cellular outcomes of RSR inactivation in retinal and lens progenitor cells leads to the question of why progenitor cells show different sensitivity to RSR inactivation. Future studies in this field might bring exciting new contributions to the understanding of the RSR and its implications for developmental syndromes.

## Author Contributions

Both authors wrote the manuscript and read and agreed to the published version of the manuscript.

## Conflict of Interest

The authors declare that the research was conducted in the absence of any commercial or financial relationships that could be construed as a potential conflict of interest.

## Publisher’s Note

All claims expressed in this article are solely those of the authors and do not necessarily represent those of their affiliated organizations, or those of the publisher, the editors and the reviewers. Any product that may be evaluated in this article, or claim that may be made by its manufacturer, is not guaranteed or endorsed by the publisher.
